# Prospects of stem cell therapy for diabetic microvascular complications

**DOI:** 10.1111/jdi.13916

**Published:** 2022-10-12

**Authors:** Keiko Naruse

**Affiliations:** ^1^ Department of Internal Medicine, School of Dentistry Aichi Gakuin University Nagoya Japan

## Abstract

The expectations for the clinical application of stem cell therapy for diabetic microvascular complications are increasing, as stem cell transplantation improves histopathological abnormalities mainly through angiogenesis/protection, nerve elongation/protection, and anti‐inflammatory effects.

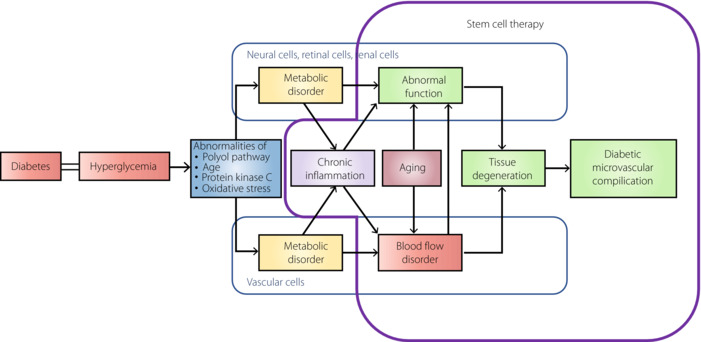

Diabetic neuropathy, nephropathy and retinopathy, which are diabetic microvascular complications, have common mechanisms of onset and progression. In addition to the four classical mechanisms of metabolic abnormalities caused by chronic hyperglycemia (increased activity of the polyol pathway, increased advanced glycation end‐products, increased oxidative stress and change of protein kinase C activity), chronic inflammation and aging affect the progression of diabetic microvascular complications. It causes blood flow disorders, including vascular endothelial cell dysfunction and tissue dysfunction. Although glycemic control is important for suppressing diabetic microvascular complications, there is a demand for more powerful therapeutic methods that can improve histopathological abnormalities.

To suppress and treat the onset and progression of diabetic microvascular complications, a therapeutic method with multifaceted effects corresponding to the complicated onset and progression mechanisms is required. In recent years, stem cell transplantation therapy is expected to be effective for various diseases, such as cardiovascular, neurological and inflammatory diseases. The main therapeutic mechanisms of stem cell transplantation are angiogenesis/vasoprotective, neuroprotective and anti‐inflammatory effects. As these therapeutic mechanisms of stem cell transplantation are consistent with the developmental mechanism of diabetic microvascular complications, its therapeutic effect can be promising.

Various cell types are used for stem cell transplantation therapy, such as bone marrow‐derived mesenchymal stem cells (MSCs), adipose‐derived MSCs, umbilical cord blood‐derived MSCs, dental pulp stem cells (DPSCs) and embryonic stem/induced pluripotent stem cell‐derived cells. In particular, MSCs, including DPSCs, are easy to extract, have excellent self‐proliferation abilities and secrete many factors. Mesenchymal cells can differentiate into mesoderm‐derived tissues, such as osteocytes, chondrocytes, muscle cells and vascular cells, as well as differentiate into ectoderm‐derived nerve cells and some endoderm‐derived hepatocytes. Allogenic MSC therapy for graft‐versus‐host disease, which utilizes the immunosuppressive effects of MSCs, has been clinically applied with good results. However, with the exception of clinical trials, stem cell therapy is still in the research stage, centered on animal experiments.

It has been found that many of the transplanted stem cells disappear from the transplanted site after a certain period. Various secretomes from stem cells at the early stages of transplantation are considered to play an important role in their effects. The results of various types of stem cell transplantation have been reported for diabetic microvascular complications. Many animal experiments have reported that stem cell transplantation improves the histological abnormalities of diabetic microvascular complications, and shows therapeutic effects through its angiogenic/vasoprotective, nerve elongation/protective and anti‐inflammatory actions (Figure 1).

**Figure 1:**
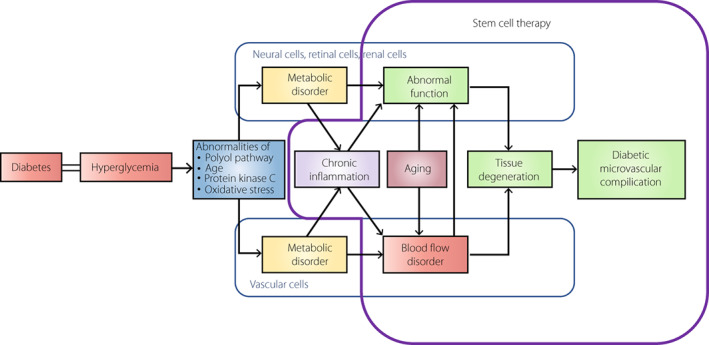
Possibility of stem cell therapy on the pathogenesis and the progression of diabetic microvascular complications. AGE, advanced glycation‐end product.

Stem cell transplantation therapy for diabetic neuropathy improves nerve conduction velocity, increases nerve blood flow and improves sensory disorders. Histological examinations have shown that stem cell transplantation improves morphological abnormalities of the sural nerves^1^. The number of monocytes/macrophages in the sciatic nerve and the expression of inflammatory macrophage‐related genes, such as tumor necrosis factor‐alpha, are increased in diabetic peripheral nerves. DPSC transplantation ameliorates chronic inflammation of the sciatic nerves^2^. Most of the administration site for transplantation is the hindlimb skeletal muscles; however, some reports have confirmed that intravenous administration is also effective to some extent. The culture supernatant of MSCs contains many secretomes, and an *in vitro* study confirmed that administration of the culture supernatant of MSCs elongates the neurites of dorsal root ganglion neurons. It has been confirmed that stem cell transplantation improves intraepidermal nerve density in diabetic models.

In diabetic nephropathy, morphological abnormalities, such as glomerular hypertrophy, podocyte damage and loss, extracellular matrix increase, mesangial cell increase, glomerular basement membrane thickening, and glomerular and interstitial fibrosis, are observed. Animal studies on stem cell transplantation for diabetic nephropathy have mainly been carried out under renal capsule or intravenously. MSC transplantation decreased serum creatinine levels and urinary microalbuminuria, as well as inflammatory cell infiltration and inflammation in the kidneys. It suppresses cytokine expression, and improves glomerular and stromal fibrosis^3^.

Stem cell transplantation for diabetic retinopathy often involves intravitreal administration. Owing to the angiogenic effect of stem cell transplantation, it is necessary to pay attention to the exacerbation of diabetic retinopathy. However, in animal experiments that carried out stem cell transplantation for diabetic retinopathy, exacerbation of diabetic retinopathy was not observed. Intravitreal transplantation of MSCs improves retinal vascular dysfunction, electroretinogram responses and retinal vascular leakage^4^. MSC transplantation also increased retinal brain‐derived neurotrophic factor levels, which were decreased in diabetic retinopathy after an increase in retinal ganglion cells.

Several methods have been investigated in stem cell transplantation, such as administering MSCs and differentiating them into neural stem cells^4^. Stem cell suspensions have been used in many cases. In diabetic nephropathy, it is reported that a method of transplanting stem cells in a sheet form under the renal capsule is also effective^5^. However, dysfunction of MSCs due to diabetes and aging adversely affects transplantation efficacy. It has been reported that transplantation after improving stem cell function with drugs or umbilical cord extracts improves the effects of stem cell transplantation^6^. The use of DPSCs, which are a type of MSCs, is another way to avoid stem cell dysfunction as a result of diabetes and aging, as DPSCs can be isolated and cultured from teeth that have been removed due to orthodontic treatment at a young age, and cryopreserved. The immunosuppressive effects of MSCs might enable allogeneic transplantation in diseases other than graft‐versus‐host disease.

As aforementioned, most effects of stem cell transplantation therapy are believed to be mediated by secretomes from transplanted cells in the early stages of transplantation. Therefore, the administration of secretomes from stem cells, such as secreted factors, extracellular vesicles and exosomal microribonucleic acids, has been investigated and reported to be a useful therapeutic method that exerts the effects of stem cell transplantation without using cells^7^, ^8^.

The expectations for the clinical application of stem cell therapy for diabetic microvascular complications are increasing, as stem cell transplantation improves histopathological abnormalities mainly through angiogenesis/protection, nerve elongation/protection and anti‐inflammatory effects. Although there are various types of stem cells, MSCs have been extensively studied for diabetic microangiopathy and their safety has been confirmed by current clinical applications. Stem cell dysfunction due to diabetes is an issue that must be resolved. Since the animal studies demonstrated that the administration of secretomes from stem cells is effective for diabetic microvascular complications, and it is hoped that this method will get closer to clinical application.

## DISCLOSURE

The author declares no conflict of interest.
